# PSMD11 and PSMD14 may serve as novel biomarkers for the prognosis of pancreatic ductal adenocarcinoma

**DOI:** 10.3389/fonc.2025.1555649

**Published:** 2025-03-20

**Authors:** Yan-Hui Yang, Zhe-Hua Xing, Hao Wang, Chi Zhang, Yu-Bo Liu, Qian-Qian Bai, Fang-Fei Liu, Wei-Feng Liu, Jun-Chuan Yang, Da-Huan Li, Hua Fan

**Affiliations:** ^1^ Department of Emergency Medicine, The First Affiliated Hospital, and College of Clinical Medicine of Henan University of Science and Technology, Luoyang, China; ^2^ The First Affiliated Hospital, and College of Clinical Medicine of Henan University of Science and Technolog, Luoyang, Henan, China; ^3^ Department of Hepatobiliary Surgery, The First Affiliated Hospital, and College of Clinical Medicine of Henan University of Science and Technology, Luoyang, China; ^4^ Office of Research & Innovation, The First Affiliated Hospital, College of Clinical Medicine of Henan University of Science and Technology, Luoyang, China

**Keywords:** PSMD11, PSMD14, poor prognosis, malignant behavior, pancreatic ductal adenocarcinoma

## Abstract

**Background:**

The ubiquitin proteasome system is involved in the regulation of cellular gene transcription and cellular receptor function through the degradation of proteins, thus affecting tumorigenesis and development. In this study, bioinformatics analysis revealed the expression of PSMD11 and PSMD14 in pancreatic ductal adenocarcinoma, which can be used as biomarkers for the prognosis of patients with PDAC. This study provides new targets for the prognostic assessment and targeted therapy of pancreatic ductal adenocarcinoma.

**Methods:**

The expression levels and prognostic value of PSMD11 and PSMD14 in pancreatic ductal adenocarcinoma patients were analyzed using the GEPIA2, GEO, TCGA and GTEx databases, and the relationships between these expression levels and clinical case data and the survival and prognosis of patients with pancreatic ductal adenocarcinoma were analyzed. The effects of PSMD11 and PSMD14 on the malignant biological behaviors of pancreatic cancer cells, such as proliferation, migration and invasion, were investigated by *in vitro* experiments.

**Results:**

Bioinformatics analysis revealed that the expression levels of PSMD11 and PSMD14 mRNAs were significantly higher in pancreatic ductal adenocarcinoma (PDAC) tissues than in normal pancreatic tissues and that this high expression was correlated with a poor prognosis in patients with PDAC. Further evaluation of the expression of PSMD11 and PSMD14 and correlation of the results with the clinical characteristics and survival of patients with PDAC revealed that high expression of PSMD11 and PSMD14 was associated with lymph node metastasis, TNM grade, degree of differentiation, and poor prognosis in patients with PDAC. Knockdown of PSMD11 and PSMD14 significantly inhibited the proliferation, migration, and invasion ability of pancreatic cancer cells.

**Conclusion:**

PSMD11 and PSMD14 are highly expressed in pancreatic ductal adenocarcinoma tissues and are correlated with the degree of malignancy of pancreatic ductal adenocarcinoma; thus, PSMD11 and PSMD14 can be used as potential prognostic biomarkers and therapeutic targets for PDAC patients.

## Introduction

Pancreatic ductal adenocarcinoma(PDAC) is a tumor disease with high lethality. Owing to the problems of difficult early diagnosis, strong invasiveness, high metastasis rates, and frequent chemotherapy resistance, the 5-year overall survival rate is less than 10%, which is the lowest among all common tumor diseases (1, 2). In recent years, the prevalence of pancreatic cancer in China has shown a significant upward trend, and at this stage, surgical treatment is preferred as the mainstay, supplemented by multidisciplinary integrated therapy with chemotherapy, targeted therapy, and immunotherapy (3). Unfortunately, however, most PDAC patients are diagnosed with advanced-stage disease. In these cases, the effect of pure surgical treatment for advanced tumors is limited, and the effects of drugs, radiotherapy, and chemotherapy are unsatisfactory and prone to recurrence and drug resistance (4, 5). Therefore, there is an urgent need to find effective prognostic markers and drug targets to improve patient survival.

The major pathways for eukaryotic protein degradation are the ubiquitin proteasome pathway (UPS) and the autophagic lysosomal system. The UPS, which is mediated by polyubiquitination of substrate proteins and degradation by the proteasome, can affect or regulate a wide range of cellular activities, including protein quality control, DNA repair, apoptosis, signal transduction, cell cycle control, gene transcription, and cellular receptor function, and it has been linked to tumorigenesis and development (6–8). The pathway consists of ubiquitinating enzymes (E1, E2, and E3), the 26S proteasome, and deubiquitinating enzymes(DUBs) and is a dynamic regulatory system for bidirectional protein modification. The proteasome is indispensable for maintaining intracellular homeostasis and is involved mainly in the degradation of polyubiquitin-labeled substrates; ubiquitinated proteins are usually recognized and degraded by the proteasome, whereas deubiquitination removes ubiquitin molecules from substrate proteins and thus maintains protein stability (9, 10).

Post-translational modifications of proteins (PTMs), including phosphorylation, ubiquitination, and acetylation, constitute a fundamental regulatory mechanism governing protein stability, localization, and functional diversity (11). These chemical modifications and their dynamic crosstalk orchestrate critical signaling networks underlying malignant transformation, tumor progression, and metastatic dissemination (12, 13). Notably, PTMs exert multifaceted control over oncogenic processes through three primary mechanisms: modulation of tumor-associated biomarker activity, reprogramming of cancer metabolism, and remodeling of the tumor microenvironment. In the human proteome, although a complete catalog of modification sites has not been established for any PTM, methods and strategies have been developed for many PTMs and hundreds of PTM sites have been identified. The analysis of PTMs has become a dynamic area in cancer research.

Numerous studies have demonstrated that the non-ATPase (PSMD) family of genes plays an integral role in biological processes through the ATP/ubiquitin pathway. The PSMD family includes many members, including PSMD1-14, which have been reported in the literature to be commonly associated with tumor development (14). Previous studies have shown that UPS dysfunction can cause a variety of diseases, including neurodegenerative disorders, malignant tumors, and cardiovascular and respiratory diseases (15, 16). When the UPS is dysfunctional, it leads to the accumulation of oncoproteins, excessive degradation of oncoproteins, impaired apoptosis, and accelerated proliferation of mutant cells, which leads to tumorigenesis. Inhibition of overactivation of the UPS may serve as a novel therapeutic approach for malignant tumors (6, 17, 18). Deubiquitinating enzyme (DUB) 26S proteasome non-ATP regulatory subunit 14 (PSMD14, also known as RPN11 and POH1) has been reported to belong to the JAMM structural domain metalloproteinase family of DUB, which is a component of the 19S regulatory cap in the 26S proteasome. PSMD14 has been demonstrated to act as an oncogene in a wide range of human cancers, e.g., ovarian cancer (19, 20), liver cancer (21), gastric cancer (22), breast cancer (23), esophageal squamous cell carcinoma (24), and lung adenocarcinoma (25, 26). Notably, the 26S proteasome non-ATPase regulatory subunit 11 (PSMD11) has been identified as an important survival factor for cancer cells. For example, small interfering RNA (siRNA)-mediated knockdown of PSMD11 triggered acute apoptosis in pancreatic cancer cells, suggesting that PSMD11 is a promising target for cancer therapy (27). It has been reported that PSMD11 affects liver cancer progression by regulating CDK4 expression and thus affects liver cancer (28). In addition, bioinformatics analysis of human blood samples revealed that PSMD11 could be used as a novel biomarker for pancreatic cancer progression, but the mechanism of its development was not further investigated (29). Therefore, the present study explored the potential of PSMD11 and PSMD14 as therapeutic targets for PDAC by interfering with their expression.

In this study, we verified that the expression of the proteasome protein PSMD11 and the deubiquitinating enzyme PSMD14 in pancreatic cancer was increased in PDAC tissues. An analysis of the relationships between the expression levels of both genes and clinicopathological features revealed that high PSMD11 and high PSMD14 were closely associated with poor patient prognosis. In addition, this study utilized the PSMD11- and PSMD14-knockdown pancreatic cancer cell lines PANC-1, MIA PaCa-2 and BxPC-3 for functional phenotyping and verified the cancer-promoting functions of PSMD11 and PSMD14 at the *in vitro* level, which may play key roles in pancreatic carcinogenesis and may be potential targets for pancreatic cancer treatment.

## Materials and methods

### Patients

We retrospectively analyzed the clinical and pathological characteristics of 73 patients who underwent surgical treatment at the First Affiliated Hospital of Henan University of Science and Technology from January 2017 to December 2022 (see **Table 1**). At the time of inclusion, patients in this study were excluded from preoperative radiotherapy, chemotherapy, or other antitumor treatments; had no history of other solid tumors in combination; or were given antitumor treatments postoperatively. For PDAC samples, patients without signs of distant organ metastases underwent standard pancreatectomy with lymph node dissection, in which matched noncancerous pancreatic tissue was sampled from the resection margins away from the original tumor site. The tissue samples were stored at -80°C immediately after surgery for subsequent mRNA isolation, protein extraction, or immunostaining. Tumor staging at the time of diagnosis was evaluated according to the American Joint Committee on Cancer guidelines (http://www.cancerstaging.org/) and was confirmed by two clinical pathologists at our institution. Permission for this study was obtained from all PDAC patients. The use of human specimens was approved by the Institutional Review Board of the First Affiliated Hospital of Henan University of Science and Technology.

**Table 1:** The expression of PSMD11 was correlated with clinicopathological features in PDAC patients.

Clinicopathological features	Cases	PSMD11 expression		^χ2^	P value
		High	Low		
Age				0.374	0.541
≤60	34	19	15		
>60	39	19	20		
Gender				0.736	0.391
Male	40	19	21		
Female	33	19	14		
Tumor size(cm)				0.096	0.757
≤4	53	27	26		
>4	20	11	9		
Lymph node metastasis				23.847	0.001^*^
No	41	11	30		
Yes	32	27	5		
TNM stge				8.324	0.004^*^
I+II	46	18	28		
III+IV	27	20	7		
Differentiation degree				16.412	0.001^*^
Well	12	5	7		
Moderate	44	20	24		
Poor	17	13	4		
Diabetes mellitus				0.145	0.704
No	57	29	28		
Yes	16	9	7		
Nerve infiltration				1.563	0.211
No	49	23	26		
Yes	24	15	9		
CA199				0.026	0.872
≤200	41	21	20		
> 200	32	17	15		

*, P<0.05. PDAC, Pancreatic ductal adenocarcinoma; PSMD11, proteasome 26S subunit, non-ATPase 11.

### Bioinformatics analysis

The data for this study were obtained from the GEO, TCGA, and GTEx databases. Three separate datasets related to pancreatic cancer are available in the GEO database: GSE28735, GSE62452, and GSE71729. GSE28735 contains 45 normal samples and 45 tumor samples, GSE62452 contains 61 normal samples and 69 tumor samples, and GSE71729 contains 46 normal samples and 145 tumor samples.

### Immunohistochemical (IHC) staining and scoring

Paraffin-embedded pancreatic cancer tissues were deparaffinized and rehydrated. Endogenous peroxidase was subsequently blocked with 3% hydrogen peroxide at room temperature for 10 min. Postclosure sections were subsequently incubated with PSMD11 antibody (Thermo Fisher, PA5-120342, 1:250) or PSMD14 antibody (Thermo Fisher, MA5-36140, 1:300) overnight at 4°C, followed by incubation with anti-rabbit/mouse HRP-labeled polymer (Proteintech, China) for 30 min at room temperature. The expression of PSMD11 and PSMD14 was detected using microscopy. Immunohistochemical (IHC) staining was scored equally: a score was calculated on the basis of the sum of the percentage of positive staining of the tumor cells: 0–5% was scored as 0, 6–35% was scored as 1, 36–70% was scored as 2, and greater than 70% was scored as 3. The intensity of the staining was assigned as follows: no staining was scored as 0, weak staining was scored as 1, moderate staining was scored as 2, and strong staining was scored as 3. The final scores were assigned to the low- or high-expression groups using cell staining positivity multiplied by staining intensity as follows: “-”0-1”, “+”2-3”, “++”4-6”, and “++++”>6; low expression was defined as a total score <4, and high expression was defined as a total score ≥4.

### Cell culture

The human pancreatic cancer cell lines PANC-1 (CL-0184), MIA PaCa-2 (CL-0627), and BxPC-3 (CL-0042) were purchased from Procell. HTERT HPNE were purchased from Kinlogix. The cells were grown in a humidified environment with 5% CO2 at 37°C. PANC-1,MIA PaCa-2 and HTERT HPNE cells were cultured in DMEM supplemented with 10% fetal bovine serum and 2.5% equine serum. BxPC-3 cells were cultured in RPMI 1640 + 10% fetal bovine serum + 2.5% horse serum. Penicillin−streptomycin was added to the various media at 1% purity.

### Transient transfection

Small interfering RNA (siRNA) and siPSMD11, siPSMD14 plasmids were chemically synthesized by Tianjin Scheves Co. One day before transfection, MIA PaCa-2 and BxPC-3 cells were cultured to 30-50% confluence in 6-well plates. The cells were transfected with Lipofectamine 3000 (Invitrogen, Carlsbad, CA) following the manufacturer’s instructions.

### Western blotting analysis

Total cell lysates were prepared in RIPA buffer, and protein concentrations were determined using a BCA assay kit (Beyotime, P0012). A standard protocol was used for the immunoblotting assay. The cell lysates were separated on a 10% glycine SDS−PAGE gel and transferred to a PVDF membrane. The membranes were blocked with 5% BSA and 0.1% Tween-20 in TBS (TBST) for 1 h at room temperature. Primary antibodies were prepared in TBST containing 5% BSA, and the cell membranes were incubated with primary antibodies overnight at 4°C. The antibodies used were purchased from Cell Signaling Technology (PSMD11, Thermo Fisher, PA5-120342, 1:2500; PSMD14, Thermo Fisher, MA5-36140, 1:2500), and the HRP-coupled secondary antibodies used were anti-mouse and anti-rabbit. The secondary antibodies were used at a concentration of 1:5000, and the bound secondary antibodies were detected using the Odyssey Imaging System.

### CCK-8 assay

The cells were inoculated into 96-well culture plates at a density of 3*10^3^ cells/well. Cell Counting Kit-8 (CCK-8) solution (10 μL, Analysis Quiz, China) was added to the culture medium. Cell proliferation was assessed at the indicated time points (0, 24, and 48h) by measuring the absorbance at 450 nm using an enzyme marker.

### Wound healing assay

After 24 h of infection or transfection, the cells that were maintained in a confluent state were treated with 1 μg/mL mitomycin C (Sigma, USA) in serum-free medium for 1 hr. The cells were subsequently scraped in a straight line along the center of the well with a 200 μL pipette tip and then washed with PBS and cultured in serum-free medium for 24 h. The cells were then incubated in serum-free medium. The wound gap was measured under a microscope at the beginning (0 h) and at the end (24 h) of the experiment.

### Transwell assay

The upper surface of the transwell chamber (Corning, USA) was precoated with Matrigel gel. A total volume of 200 μL of serum-free RPMI 1640 medium containing 1 × * 10^5^ cells was added to the upper chamber (3 × 10^4^ cells/well). The lower chamber was supplemented with DMEM containing 10% FBS as an elicitor. After incubation at 37°C and 5% CO2 for 24 h, the cells invading the lower chamber were fixed with 4% paraformaldehyde for 20 min and stained with 0.5% crystal violet for 5 min. Finally, the number of cells was recorded under a microscope.

### Follow-up

The postoperative follow-up included clinical examination and laboratory tests. Overall survival was a prognostic measure defined as the time from the date of surgery to the date of death or the last follow-up examination.

### Statistical analysis

All the data are expressed as the means ± SDs. All the statistical analyses were performed using SPSS 24.0 and GraphPad Prism 5 software. The relationships between PSMD11 and PSMD14 expression and clinicopathological features were analyzed via the chi-square test and Fisher’s exact probability method. Survival curves were evaluated using the Kaplan−Meier method, and differences between survival curves were tested using the log-rank test. Univariate and multivariate Cox regression analyses were used to evaluate variables associated with overall survival in patients with PDAC. In all the statistical analyses, statistical significance was achieved when p<0.05.

## Results

### Transcript abundance and prognostic significance of PSMDs in pancreatic cancer patients

**Figure 1:**
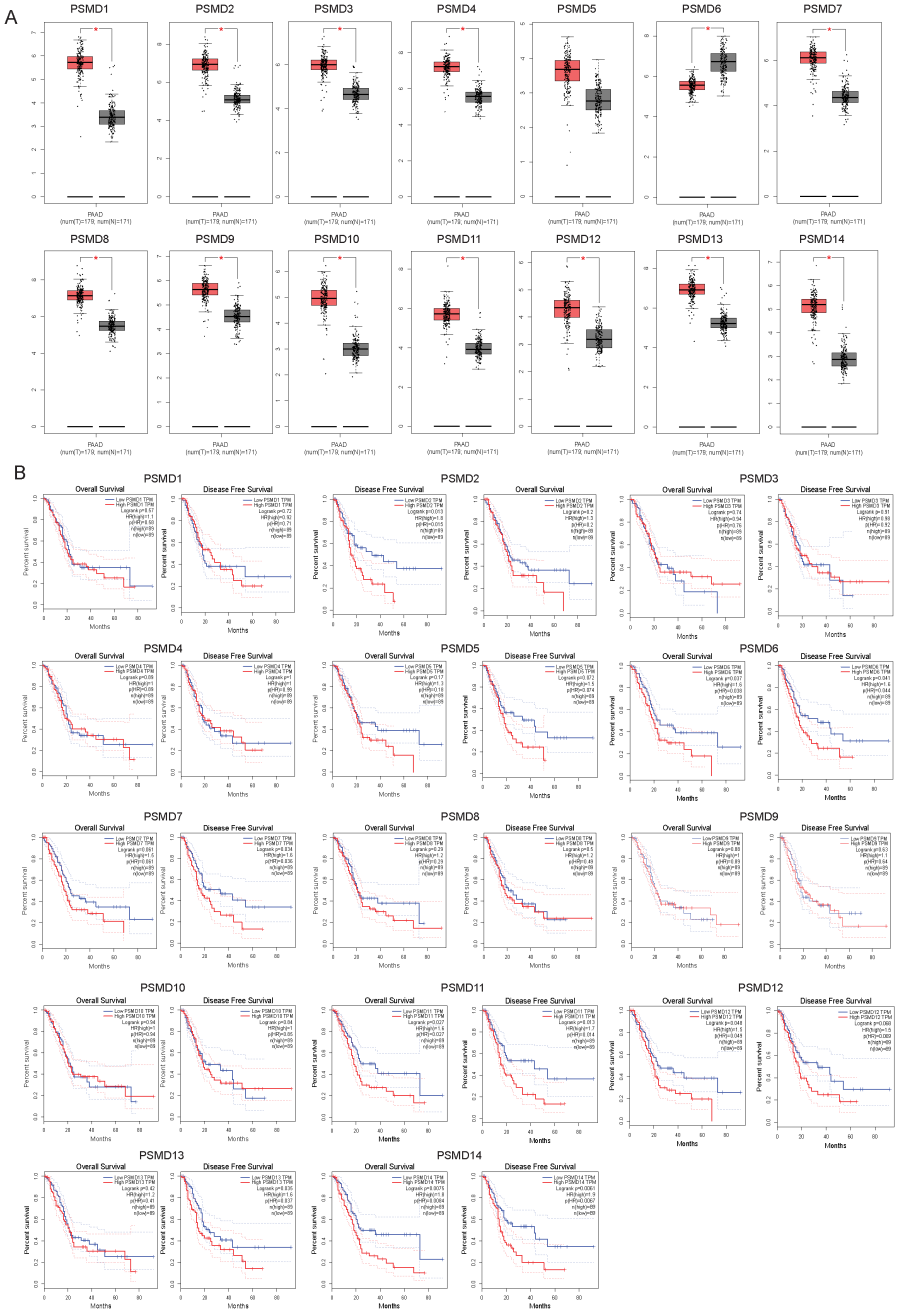
Transcript abundance and prognostic significance of PSMDs in pancreatic cancer patients. **(A)** The mRNA levels of PSMD1-14 in pancreatic cancer and normal samples from GEPIA2. PAAD, pancreatic adenocarcinoma; T, tumor samples; N, normal samples. **(B)** The relationship between the mRNA levels of PSMD1-14 and the overall and disease-free survival of pancreatic cancer patients.

To elucidate the clinical implications of proteasome 26S subunit non-ATPase (PSMD) family members in pancreatic cancer pathogenesis, we performed comprehensive transcriptomic analysis using the GEPIA database. Systematic evaluation of PSMD1-14 expression revealed significant upregulation (p<0.05) of PSMD1, PSMD2, PSMD3, PSMD4, PSMD7-PSMD14 transcripts in pancreatic tumor tissues compared with adjacent normal controls, with the notable exception of PSMD6 which demonstrated reduced mRNA expression (**Figure 1A**). Subsequent Kaplan-Meier survival analysis identified three PSMD members (PSMD6, PSMD11, and PSMD14) exhibiting statistically significant correlations (p<0.05) with patient outcomes (**Figure 1B**). Intriguingly, elevated PSMD6 expression showed a negative association with overall survival, contrasting with the trends for PSMD11 and PSMD14. Based on these, we prioritized PSMD11 and PSMD14 for subsequent mechanistic investigations.

### Expression of PSMD11 and PSMD14 in pancancer and pancreatic cancer

**Figure 2:**
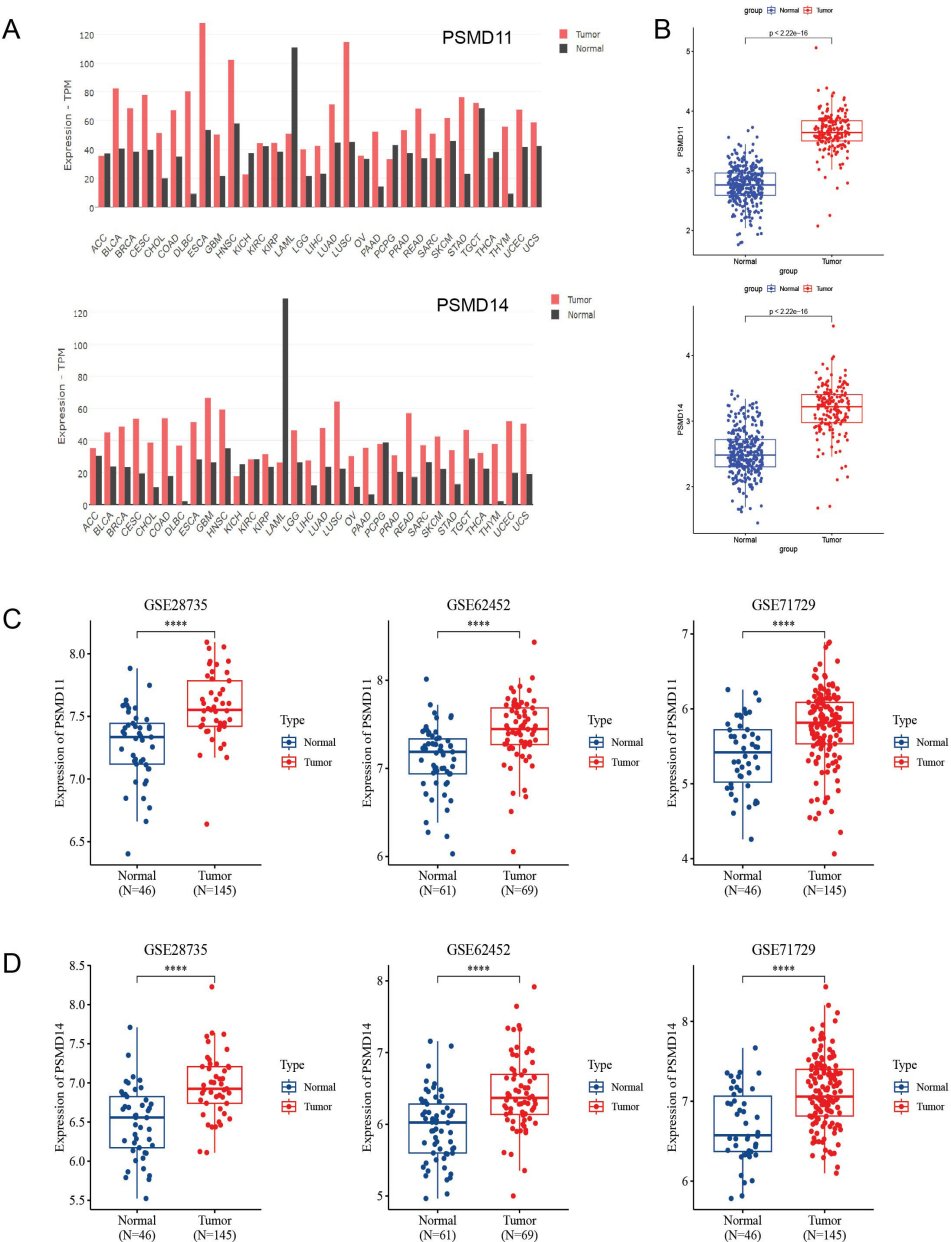
Expression analysis of PSMD11 and PSMD14 in databases. **(A)** The GEPIA2 database was used to analyze the expression levels of PSMD11 and PSMD14 in different types of tumors. **(B)** Relative expression levels of PSMD11 and PSMD14 in TCGA and GTEx databases (N = 332, T = 179). **(C)** Expression of PSMD11 in GSE 28735 (N = 46, T = 145), GSE 62452 (N=61, T=69) and GSE 71729 (N=46, T=145). **(D)** Expression of PSMD14 in GSE 28735 (N = 46, T = 145), GSE 62452 (N=61, T=69) and GSE 71729 (N=46, T=145), ****P value < 0.001.

First, we examined the expression of PSMD11 and PSMD14 in various normal and tumor tissues using the GEPIA2 online database(http://gepia2.cancer-pku.cn/). Our analysis revealed significant upregulation of PSMD11 and PSMD14 in several tumors, including pancreatic cancer (**Figure 2A**). Since relatively few TCGA normal samples are available, we investigated the mRNA expression profiles of PSMD11 and PSMD14 in the TCGA and GTEx cohorts. We found that PSMD11 and PSMD14 mRNA expression was significantly upregulated in pancreatic cancer tissues compared with normal tissues (**Figure 2B**). In addition, by analyzing the expression of PSMD11 and PSMD14 in the tumor and normal groups in the three GEO cohorts, we found that all of them were significantly different and that the expression of PSMD11 and PSMD14 was higher in the tumor group than in the normal group (**Figures 2C, D**). In conclusion, the mRNA expression levels of PSMD11 and PSMD14 were significantly higher in pancreatic cancer tissues compared with normal pancreatic tissues.

### The potential prognostic value of pancreatic cancer

**Figure 3:**
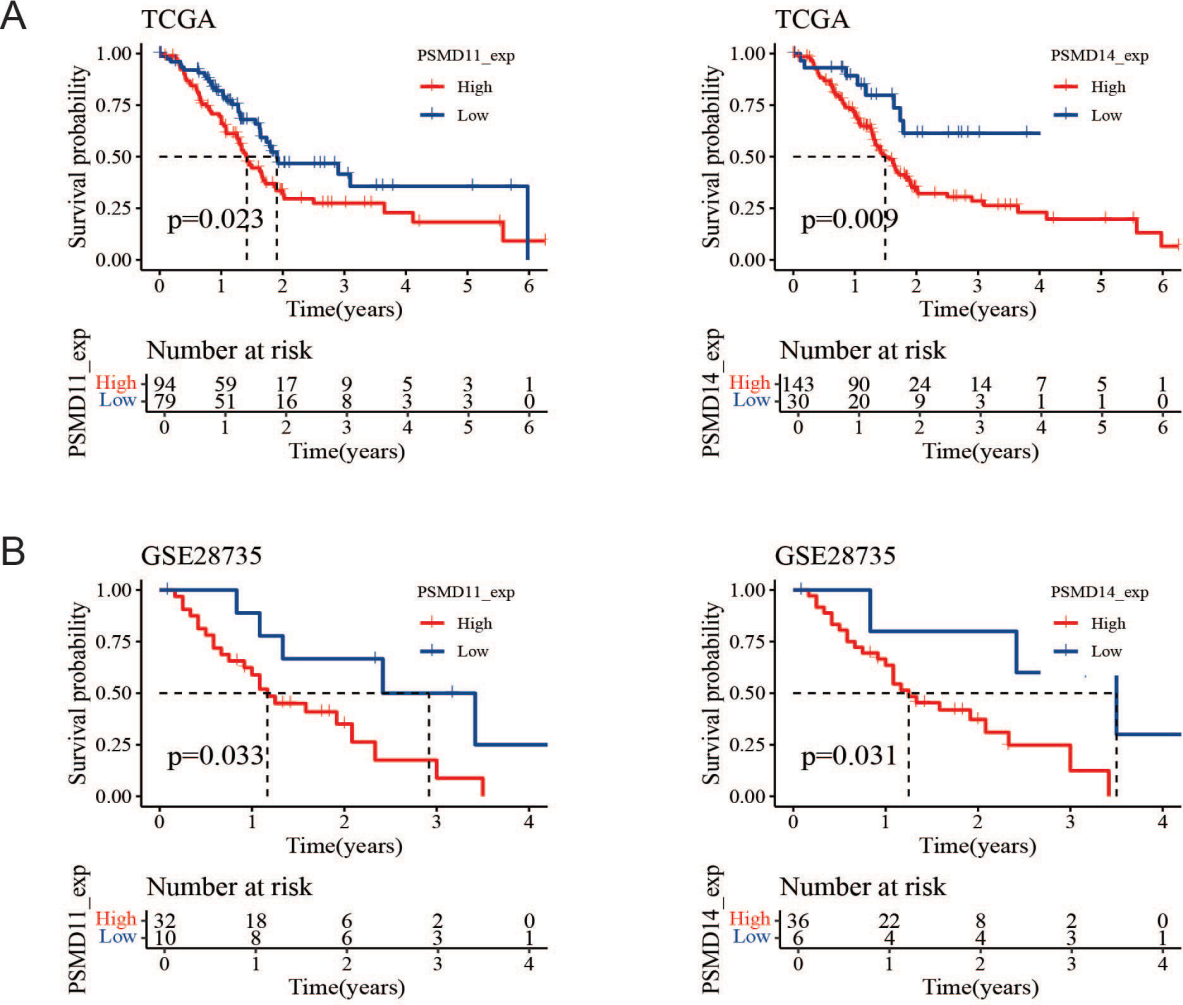
Prognostic value of PSMD11 and PSMD14 in the database. **(A)** Survival and prognosis analysis of PSMD11 and PSMD14 in TCGA data. **(B)** Survival and prognosis analysis of PSMD11 and PSMD14 in GSE28735 database.

Since the expression of PSMD11 and PSMD14 in pancreatic cancer tissues was significantly higher than that in normal tissues, we hypothesized that PSMD11 and PSMD14 might have diagnostic and prognostic value in pancreatic cancer. Notably, we further analyzed the correlations between PSMD11 and PSMD14 expression and the prognosis of patients with pancreatic cancer in the TCGA and GSE28735 databases and divided the tumor samples into high- and low-expression groups according to the optimal segmentation point. K−M curves revealed that the prognosis was worse for patients in the high-PSMD11 and PSMD14 expression groups (P<0.05) (**Figures 3A, B**).

### Associations of PSMD11 and PSMD14 expression with PDAC patients

**Figure 4:**
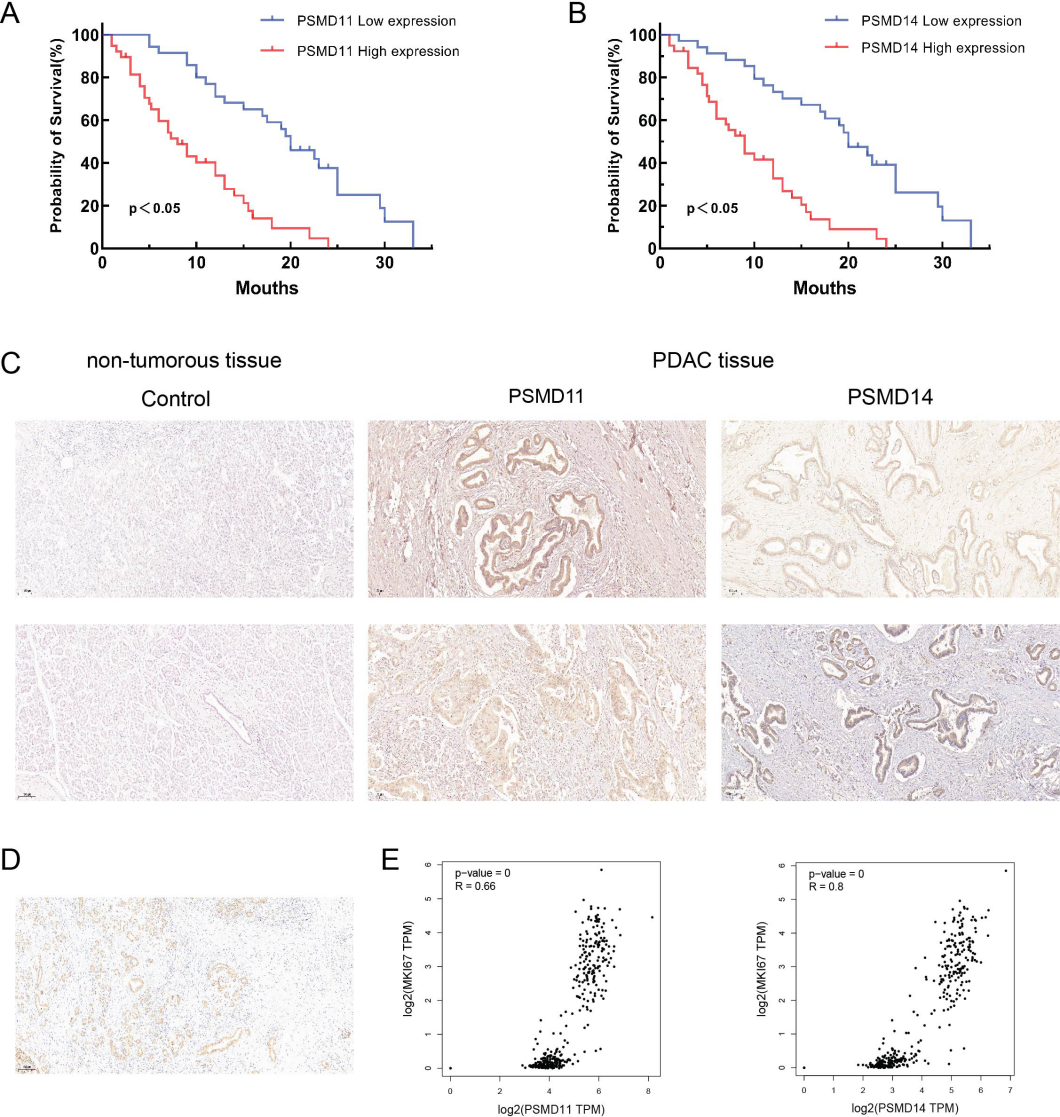
Relationship between PSMD11, PSMD14 and clinical characteristics of PDAC patients. **(A)** Relationship between PSMD11 expression and prognosis of PDAC patients; **(B)** Relationship between PSMD14 expression and prognosis of PDAC patients; **(C)** Comparison between pancreatic normal tissues and pancreatic tumor tissues (X40); **(D)** Expression of Ki-67 in PDAC tissues; **(E)** Analysis of the expression of PSMD11 and PSMD14 by the GEPIA2 database was positively correlated with the Ki-67 expression.

To elucidate the expression patterns of PSMD11 and PSMD14 in PDAC samples, we used immunohistochemical analysis to assess PSMD11 and PSMD14 expression in 73 pairs of PDAC tissues. The results revealed that the expression of both genes was significantly correlated with lymph node metastasis, TNM grade, and degree of differentiation, but the expression of both genes was not associated with patient age, sex, tumor size, lymph node metastasis, degree of differentiation, diabetes mellitus, neural infiltration, or CA199 (**Tables 1**, **2**). We subsequently analyzed the K−M curves of PSMD11 and PSMD14 to show that the poorer prognosis of the high-expression group was closely related, which was consistent to the previous analysis in the database (**Figures 4A, B**). We performed both univariate and multivariate Cox regression analyses, and the results revealed that PSMD11 and PSMD14 were independent predictors of patients’ overall survival (**Tables 3**–**5**). Therefore, both PSMD11 and PSMD14 expression may serve as prognostically relevant markers for pancreatic cancer. The expression of PSMD11 and PSMD14 was significantly elevated in malignant cells compared with normal ductal cells (**Figure 4C**), and we found that the expression of PSMD11 and PSMD14 was associated with Ki-67, a marker protein for cell proliferation (**Figure 4D**). Linear regression analysis confirmed that the expression of PSMD11 and PSMD14 in PDAC tissues was positively correlated with the expression of Ki-67 (**Figure 4E**). These results suggested that high expression of PSMD11 and PSMD14 might be associated with the malignant progression of PDAC.

**Table 2:** The expression of PSMD14 was correlated with clinicopathological features in PDAC patients.

Clinicopathological features	Cases	PSMD14 expression		^χ2^	P value
		High	Low		
Age				0.154	0.694
≤60	34	19	15		
>60	39	20	19		
Gender				0.088	0.766
Male	40	22	18		
Female	33	17	16		
Tumor size(cm)				1.483	0.223
≤4	53	26	27		
>4	20	13	7		
Lymph node metastasis				13.970	0.001*
No	41	14	27		
Yes	32	25	7		
TNM stge				17.370	0.001*
I+II	46	16	30		
III+IV	27	23	4		
Differentiation degree				18.043	0.001*
Well	12	3	9		
Moderate	48	24	20		
Poor	17	12	5		
Diabetes mellitus				0.097	0.756
No	57	31	26		
Yes	16	8	8		
Nerve infiltration				1.183	0.277
No	49	24	25		
Yes	24	15	9		
CA199				0.982	0.322
≤200	41	24	17		
>200	32	15	17		

*, P<0.05. PDAC, Pancreatic ductal adenocarcinoma; PSMD14, proteasome 26S subunit, non-ATPase 14.

**Table 3:** Univariate Cox regression analysis of prognostic factors of survival in PDAC patients.

Characteristics	Hazard ratio (95% CI)	P value
Age	1.312 (0.776-2.217)	0.311
Gender	1.115 (0.653-1.902)	0.691
Tumor size	1.376 (0.782-2.422)	0.268
Lymph node metastasis	1.565 (0.927-2.642)	0.094
TNM stge	2.433 (1.391-4.257)	0.002^*^
Diabetes mellitus	1.433 (0.792-2.593)	0.234
Nerve infiltration	0.805 (0.451-1.437)	0.805
CA199	0.990 (0.582-1.686)	0.971
PSMD11	3.923 (2.173-7.083)	0.001^*^
PSMD14	3.849 (2.118-6.995)	0.001^*^

*, P<0.05. PDAC, Pancreatic ductal adenocarcinoma; CI, confidence interval; PSMD11, proteasome 26S subunit, non-ATPase 11; PSMD14, proteasome 26S subunit, non-ATPase 14.

**Table 4:** Multivariate Cox regression analysis of prognostic factors of survival in PDAC patients.

Characteristics	Hazard ratio (95% CI)	P value
Lymph node metastasis	0.635 (0.329-1.226)	0.176
TNM stge	2.023 (1.053-3.886)	0.031*
PSMD11	4.071 (2.093-7.917)	0.001*

*, P<0.05. PDAC, Pancreatic ductal adenocarcinoma; CI, confidence interval; PSMD11, proteasome 26S subunit, non-ATPase 11.

**Table 5:** Multivariate Cox regression analysis of prognostic factors of survival in PDAC patients.

Characteristics	Hazard ratio (95% CI)	P value
Lymph node metastasis	0.813 (0.434-1.523)	0.518
TNM stge	1.487 (0.755-2.928)	0.251
PSMD14	3.521 (1.771-6.999)	0.001*

*, P<0.05. PDAC, Pancreatic ductal adenocarcinoma; CI, confidence interval; PSMD14, proteasome 26S subunit, non-ATPase 14.

### Knockdown of PSMD11 and PSMD14 reduces PDAC cell proliferation, invasion and migration

**Figure 5:**
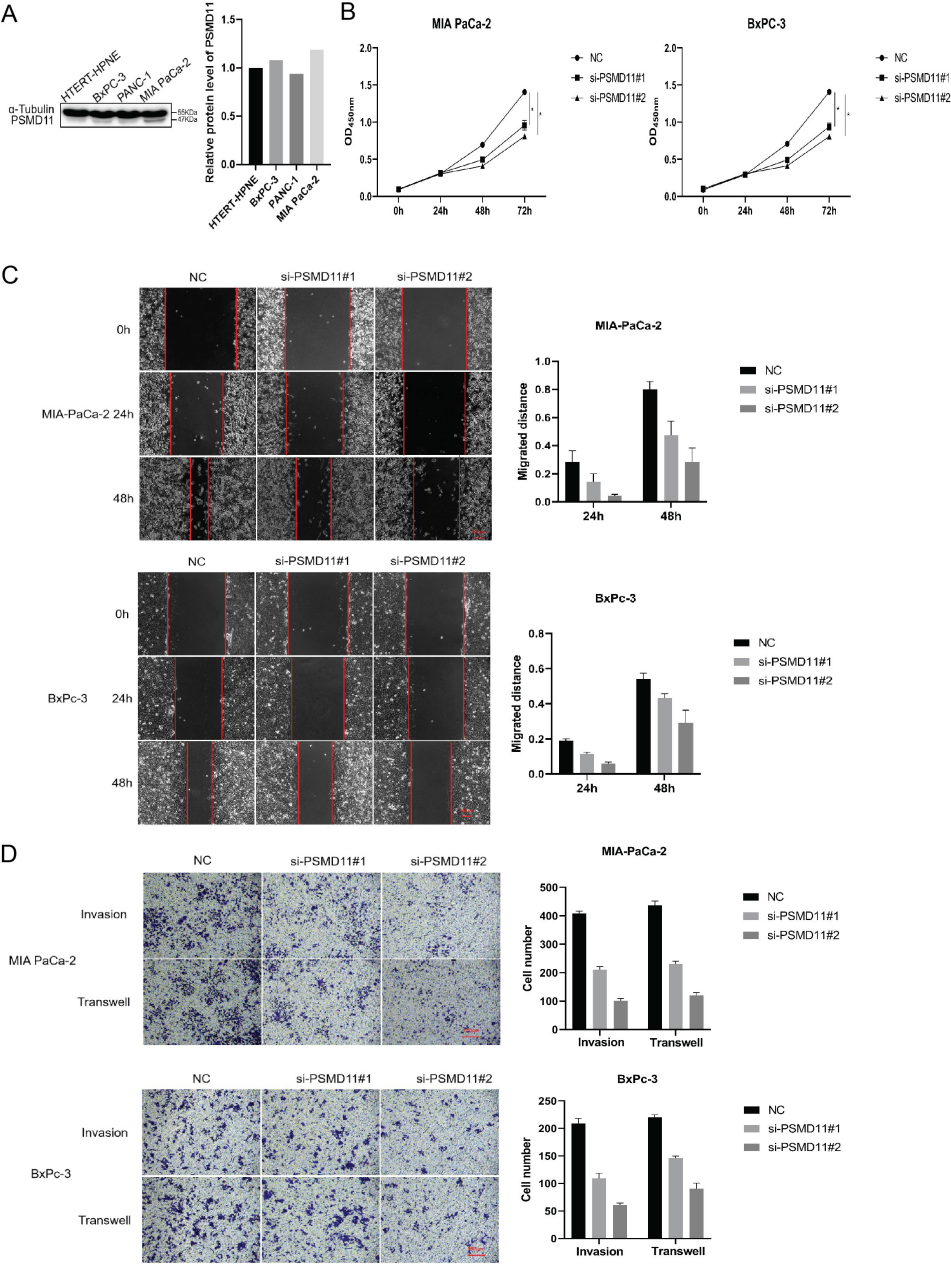
Effect of PSMD 11 gene knockout on pancreatic cancer cell growth. **(A)** Expression of PSMD11 in pancreatic cancer cell lines and pancreatic cells; **(B)** Cell viability was determined by CCK-8 assay; **(C)** Cell migration was assessed with a scratch assay. **(D)** Cell invasion was determined by the Transwell method. Each experiment is performed three times.

**Figure 6:**
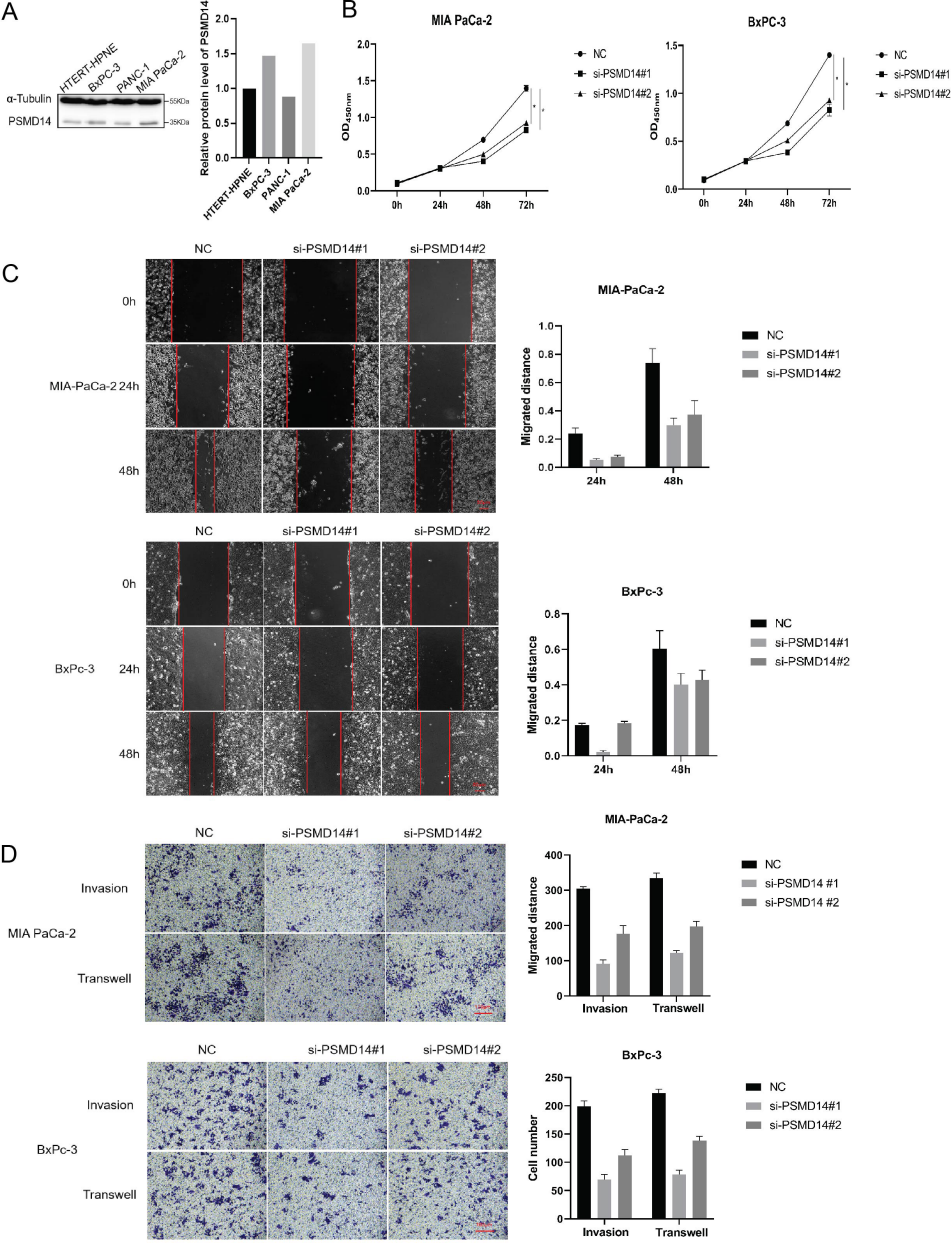
Effect of PSMD 14 gene knockout on pancreatic cancer cell growth. **(A)** Expression of PSMD14 in pancreatic cancer cell lines and pancreatic cells; **(B)** Cell viability was determined by CCK-8 assay; **(C)** Cell migration was assessed with a scratch assay. **(D)** Cell invasion was determined by the Transwell method. Each experiment is performed three times.

To investigate the functions of PSMD11 and PSMD14 in pancreatic cancer, We measured the expression levels of pancreatic cancer cell lines and pancreatic cell (**Figure 5A**). We stably infected MIA PaCa-2 and BxPC-3 cells with PSMD11 shRNA. The protein level of PSMD11 in PSMD11-knockdown cells was verified by Western blotting. A CCK-8 assay was performed to assess cell viability, and the results revealed that PSMD11 knockdown significantly reduced the proliferative capacity of MIA PaCa-2 and BxPC-3 cells (**Figure 5B**). Next, we examined the effects of PSMD11 on pancreatic cancer migration and invasion *in vitro*. PSMD11 knockdown inhibited the migration of MIA PaCa-2 and BxPC-3 cells (**Figure 5C**). Compared with the control, PSMD11 knockdown reduced the invasive ability of MIA PaCa-2 and BxPC-3 cells (**Figure 5D**). In addition, we performed the above experiments on PSMD14 and reported that PSMD14 has the same function as PSMD11 (**Figures 6A–D**). In summary, PSMD11 and PSMD14 could promote the proliferation, invasion, and migration of PDAC cells.

## Discussion

Pancreatic ductal adenocarcinoma accounts for more than 90% of malignant pancreatic tumors, and its characteristics of being undetectable, highly lethal, and invasive have led to significant challenges in the early diagnosis and treatment of PDAC (30). At the time of diagnosis, most patients are in the middle to late stages of the disease, lack effective therapies and have extremely poor prognoses. Therefore, there is an urgent need to develop new methods for the early diagnosis and effective treatment of pancreatic cancer. With global research on multiomics and gene sequencing, people have gained a deeper understanding of the molecular mechanism of pancreatic cancer, more signaling pathways and targets have been discovered, and an increasing number of targeted therapies and immunotherapies have been developed (31). The treatment mode of pancreatic cancer is gradually shifting from traditional surgical resection to more precise individualized treatment, which has introduced a new dawn to the treatment of pancreatic cancer. As a promising therapeutic method, targeted therapy has made significant research progress in the field of pancreatic cancer treatment in the past 20 years (32).

The ubiquitin–proteasome system and autophagy are the two major mechanisms of intracellular protein degradation, which mainly maintain intracellular protein stability by timely degradation of folded, damaged and unwanted proteins. However, in nature, the ubiquitin proteasome system is a representative process of protein degradation and is involved in the degradation of 80% of intracellular proteins, which is a multistep reaction process that includes ubiquitin activation, binding and conjugation, and proteasome recognition and degradation (33). Our preliminary study of autophagy revealed that autophagy plays a tumor-promoting role in pancreatic cancer and hepatocellular carcinoma, and further study revealed that autophagy and the ubiquitin proteasome system mutually regulate one another’s function: autophagy can be induced by inhibiting the expression of the proteasome, and the inhibition of autophagy can also cause short-term protein aggregation (34). The expression profiling of pancreatic cancer genes was later performed by using LC3 as a guideline, and differential genes containing several key proteins of the ubiquitin proteasome system (PSMD11, PSMD14, etc.) that have not yet been clearly associated with autophagy processes in human pancreatic cancer cells were finally identified. However, the relationship between the autophagy-related lysosomal system and the ubiquitin–proteasome system is still unclear. Therefore, the aims of the present study were to explore how the ubiquitin–proteasome system played a role in pancreatic cancer and to provide a theoretical basis for further studies on the interconnection between the ubiquitin–proteasome system and the autophagy−lysosome system.

In a previous study, knockdown of PSMD11 effectively inhibited the proliferation and apoptosis of hepatocellular carcinoma cell lines and promoted their proliferation by regulating the ubiquitination and enhancing the protein stability of CDK4 (35). High expression of PSMD11 is significantly correlated with overall survival in patients with gastric cancer (36). PSMD14 modulates the progression of breast cancer through ERα signaling (37). PSMD14 inhibits autophagy and thus influences the progression of ovarian cancer through the LRPPRC/Beclin1-Bcl-2/SQSTM1 signaling pathway (20). The Beclin1-Bcl-2/SQSTM1 signaling pathway inhibits autophagy and thus affects ovarian cancer progression. In another study, PSMD14 was differentially expressed in gastric cancer and promoted GC progression by stabilizing PTBP1 (23). PSMD14 is strongly associated with poor prognosis in lung adenocarcinoma (LUAD), and PSMD14 knockdown significantly inhibits cell growth and affects lung cancer progression by modulating p21 stability, leading to G1-phase arrest and cellular senescence (26). Collectively, our mechanistic investigations and clinical data analyses establish that PSMD11 and PSMD14, as critical regulatory subunits of the ubiquitin-proteasome system (UPS), drive oncogenic progression through targeted degradation of tumor-suppressive proteins and stabilization of oncoproteins. This pathophysiological mechanism has been experimentally validated across multiple cancer types (38), but their involvement in pancreatic cancer has not been elucidated.

In this study, the expression analysis of PSMD11 and PSMD14 across cancers revealed that they were differentially expressed in a variety of tumors, including pancreatic ductal adenocarcinoma, and analysis using the GEO database revealed that the expression levels of these two proteins were significantly higher in pancreatic ductal adenocarcinoma tissues than in normal pancreatic tissues. Further analysis of patient prognosis revealed that high expression of PSMD11 and PSMD14 was closely associated with a poor prognosis in PDAC patients. Based on the information collected for our pancreatic ductal adenocarcinoma patients and immunohistochemical staining on their pathological sections, we showed that PSMD11 and PSMD14 were mainly expressed in the cytoplasm. Analysis of the patients’ clinical data revealed that the expression of both PSMD11 and PSMD14 was significantly correlated with lymph node metastasis, TNM grade, degree of differentiation, and poor prognosis in pancreatic ductal adenocarcinoma patients. Moreover, it was not associated with tumor size, CA199 level or other common pancreatic cancer clinical features. Consequently, we hypothesized that ubiquitin proteasome-associated proteins mainly affect the survival prognosis of patients with pancreatic ductal adenocarcinoma and can be further investigated as prognostic markers; however, the evidence for their use as diagnostic markers is not yet sufficient. While CA199 remains a cornerstone biomarker for pancreatic cancer diagnosis and prognosis (39), however, studies have shown that they each play different molecular roles in tumor progression. Specifically, PSMD11 and PSMD14 function as regulatory components of the ubiquitin-proteasome system (UPS), mediating substrate-specific protein degradation. In contrast, CA199 is a sialylated Lewis blood group antigen - exerts its oncogenic effects through glycan-mediated cell adhesion and metastasis potentiation. Our analysis demonstrated no statistically significant association between PSMD11/PSMD14 expression and CA199 levels in this cohort. However, as a single-center retrospective study with a limited sample size (n=73) that may compromise statistical power. To verify the effects of both PSMD11 and PSMD14 in pancreatic cancer, we confirmed that the knockdown of PSMD11 and PSMD14 could inhibit the malignant biological behaviors of pancreatic cancer cells, such as their proliferation, migration, and invasion, via the MTT assay, cell scratch assay, and transwell assay, which suggested that these two proteins could be used as potential markers for pancreatic cancer progression and prognostic assessment. In addition, they may also function as potential therapeutic targets.

This study further clarified the role of PSMD11 and PSMD14 in the occurrence and development of pancreatic ductal adenocarcinoma. However, this study has several limitations. First, it is a retrospective analysis based on clinical cases, which introduces the possibility of selection bias and incomplete information. Therefore, further prospective studies, multicenter studies, and randomized controlled trials are necessary to validate the results. Second, this study relies heavily on the analysis of gene expression and correlation with clinical data. In-depth cellular functionality experiments and animal modeling studies remain to be conducted, and further experimental studies are needed to validate the findings of this study. Future research directions may include the following: first, further study of the detailed mechanisms of deubiquitinating enzymes and ubiquitinosomes in pancreatic cancer development, especially in cell cycle regulation, cell proliferation and apoptosis-related pathways, is needed. Second, the role of PSMD11 and PSMD14 in pancreatic cancer metastasis and prognosis, and their potential value in individualized therapy, merit further exploration. Finally, the interactions of PSMD11 and PSMD14 with other molecules were explored by studying other related genes and signaling pathways to gain a deeper understanding of the mechanisms of pancreatic carcinogenesis. In conclusion, this study revealed the important roles of PSMD11 and PSMD14 in pancreatic cancer and provided insights into their underlying mechanisms. These findings will contribute to a better understanding of the pathogenesis and prognostic assessment of pancreatic cancer.

## Data Availability

The datasets presented in this study can be found in online repositories. The names of the repository/repositories and accession number(s) can be found in the article/supplementary material.
